# Successful Empirical Treatment of Suspected Spinal Tuberculosis: A Case Report

**DOI:** 10.7759/cureus.55562

**Published:** 2024-03-05

**Authors:** Yusoff Norisyam, Jaya Thilak Shanmugam, Han Sim Lim, Zairul Bahrin

**Affiliations:** 1 Orthopedics, Hospital Pulau Pinang, George Town, MYS; 2 Spine Surgery, Hospital Pulau Pinang, George Town, MYS

**Keywords:** negative tissue biopsy, antitubercular therapy, cervicothoracic, empirical treatment, spinal tuberculosis

## Abstract

Spinal tuberculosis is an uncommon extrapulmonary manifestation of tuberculosis infection, known as a great masquerade that often mimics other pathologies, such as pyogenic and non-pyogenic infection, bone metastasis, haematological malignancy, and metabolic bone disease. It presents great challenges in establishing a diagnosis, deciding on treatment, and monitoring the response to treatment. A tissue-proven diagnosis is the cornerstone of a definitive diagnosis before initiating medical antitubercular therapy, leading to successful treatment. Here, we present a distinct and rare instance of spinal tuberculosis with an atypical presentation of upper thoracic myelopathy. It involved the cervicothoracic junction, exhibiting minimal axial symptoms but intensive destruction of the affected levels radiologically, along with an incomplete neurological deficit and the possibility of catastrophic neurological complications. The ultimate distinctiveness of this case lies in the diagnostic challenge it posed. Despite undergoing three separate tissue biopsies, tuberculosis infection could not be established, as all results returned negative for cellular, molecular, and histopathological markers, leading to a delay in initiating empirical medical therapy. Nonetheless, the patient responded well to empirical antitubercular therapy, resulting in favourable outcomes. To the best of our knowledge, a case of spinal tuberculosis with numerous negative tissue diagnoses has not been previously reported.

## Introduction

Extrapulmonary tuberculosis represents 10% of all tuberculosis cases, with half involving the musculoskeletal system [1]. The spine is the most common musculoskeletal site, accounting for between 1% and 2% of all tuberculosis cases, with the thoracolumbar junction being the most affected region of the spinal column, followed by the lumbar and cervical spine [2]. Extrapulmonary tuberculosis often presents as a diagnostic challenge due to nonspecific clinical findings and non-pathognomonic radiological features. It poses therapeutic challenges due to difficulties associated with accessing the site of involvement and assessing therapeutic response. Additionally, the impact of prolonged antituberculosis treatment is significant. Furthermore, extrapulmonary tuberculosis has significant health implications, leading to disability secondary to instability, pain, deformity, and neurological complications. Therefore, it is crucial to promptly establish a definitive tissue-proven diagnosis before initiating medical therapy, which has a well-established good response in the literature. Surgical intervention is indicated in cases of diagnostic uncertainty and poor response to medical therapy, especially in the presence of complications, such as neurological deficits, significant physiological instability, and worsening spinal deformity.

## Case presentation

We present a case of a 13-year-old boy, previously bacillus Calmette-Guérin (BCG) vaccinated, who presented with gradually worsening bilateral lower limb weakness and numbness over the past three months, associated with stiffness and involuntary movement of the bilateral lower limbs. The symptoms progressively worsened in the past two weeks, leading to an unstable gait. He became unable to ambulate due to instability and stiffness, necessitating the use of a wheelchair for mobility. Otherwise, he had normal bowel and bladder function, minimal upper back pain with no rest pain or night pain, no radicular pain, and a pain score that did not affect his daily activities.

In the two months preceding the presentation, the patient developed generalized body weakness with a loss of appetite and a weight loss of 10 kg from baseline. Despite this, there was no fever, night sweats, chronic cough, haemoptysis, contact with a tuberculosis patient, trauma, or family history of malignancy. Additionally, there were no other joint pains, no neck pain, normal hand function with hand clumsiness, and no notice of swelling or mass elsewhere in the body. Other systemic reviews were unremarkable.

Upon examination, he was afebrile, and his vital signs were stable. The upper back examination revealed no midline tenderness without any noticeable swelling or deformity and a good range of motion in the neck. A neurological examination indicated full strength and intact sensation in bilateral upper and lower limbs. Surprisingly, the bilateral lower limbs revealed a significant upper motor neuron lesion with hyperreflexia, hypertonia, clonus, and a positive Babinski’s sign bilaterally. His upper limbs were normotonic with normal reflexes and negative myelopathic signs, and he had an intact per rectal examination.

Both cervical and inguinal lymph node examinations were non-tender, and no palpable masses were found. Respiratory examination revealed normal air entry with no additional sounds, and the remainder of the systemic examination was unremarkable.

Diagnostic assessment

Baseline blood investigations revealed a normal full blood count, except for mild anaemia with a haemoglobin level of 10.2 g/dL, along with normal total white cell count, platelet count, coagulation profiles, liver, and renal function. There were no abnormal cells in her blood picture or bone marrow.

Blood infection parameters indicated an elevated C-reactive protein (CRP) level of 60 mg/dL and a raised erythrocyte sedimentation rate (ESR) with a reading of 102 mm. Serial blood cultures and urine cultures were negative. Basic tuberculosis workup was negative for the Mantoux test and sputum acid-fast bacilli (AFB), and human immunodeficiency virus was excluded through serology.

**Figure 1:**
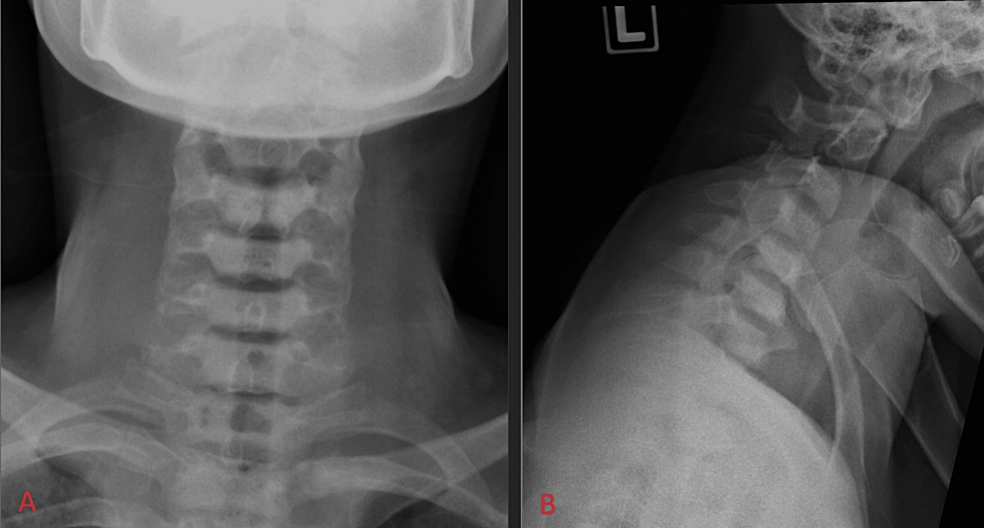
Anteroposterior (A) and lateral (B) views of plain radiography of the cervical showed normal alignment with no destruction of vertebral bodies and normal intervertebral disc spaces.

His chest radiograph was normal. Plain radiography of the cervical and thoracic regions showed normal alignment with no destruction of vertebral bodies and normal intervertebral disc spaces (Figure 1). However, the assessment of the cervical-thoracic junction was inadequate due to poor penetration by the shoulder shadow on the plain radiograph.

**Figure 2:**
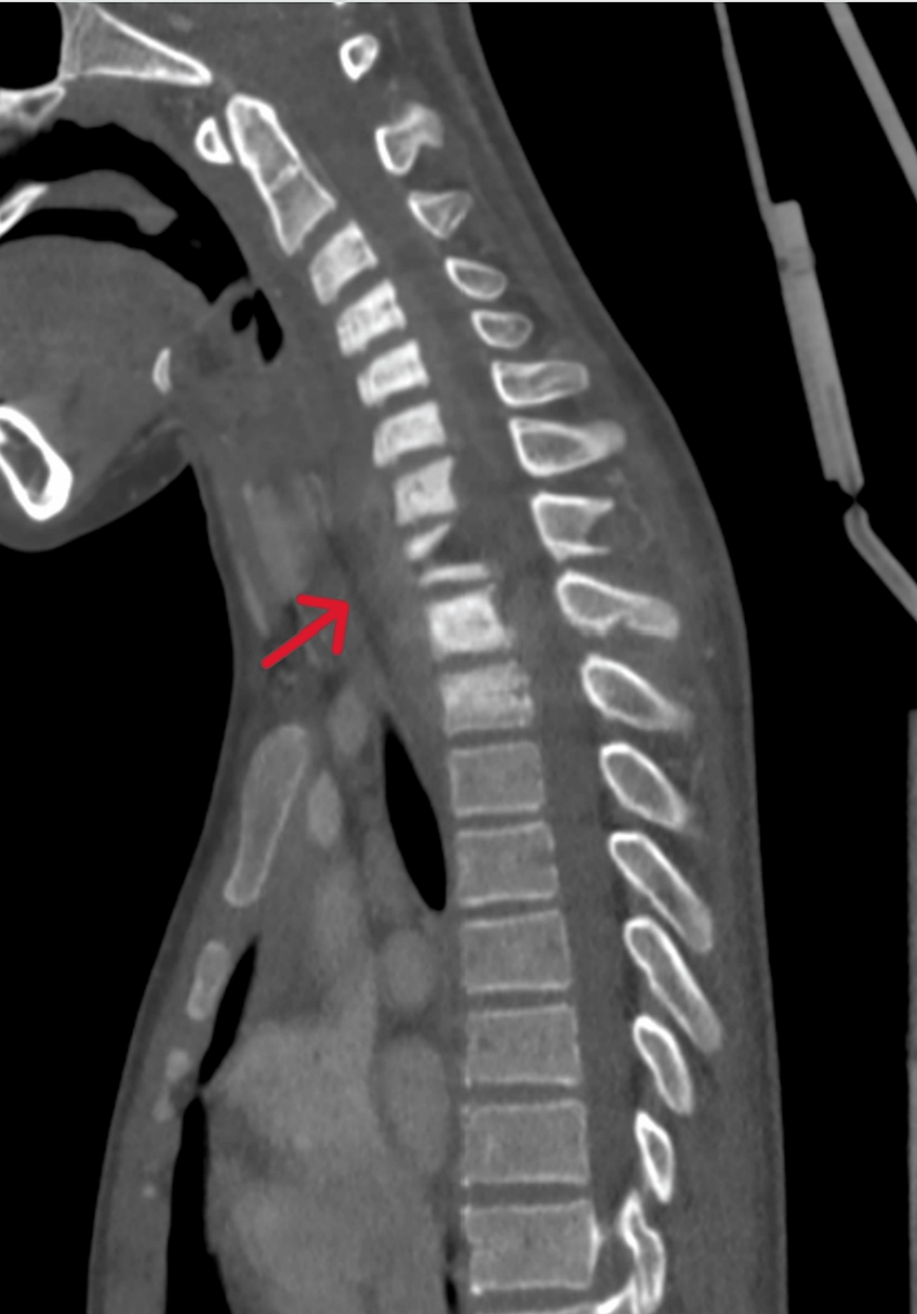
Mid-sagittal view of the CT scan of the cervicothoracic spine with a red arrow indicating the destruction of the body and spinous process of T1, along with sclerotic bony changes from the lower cervical spine down to T2 and an increased prevertebral soft tissue shadow.

A computed tomography (CT) scan of the cervical and thoracic spine (Figure 2) revealed the destruction of the body and spinous process of T1 with sclerotic bony changes of the lower cervical spine down to T2 and an increased prevertebral soft tissue shadow.

**Figure 3:**
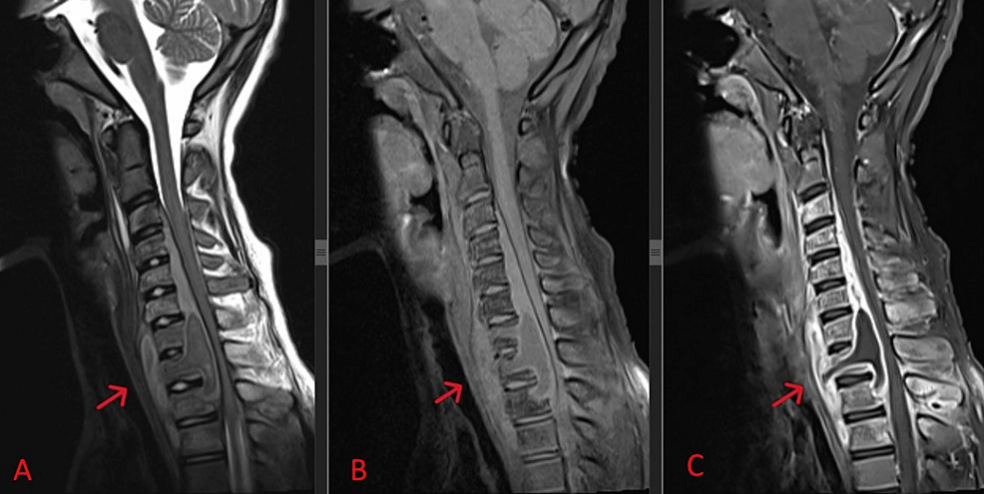
Mid-sagittal view of T2-weighted (A), T1-weighted (B), and contrast-enhanced (C) MRI of the cervicothoracic spine, with a red arrow indicating the destruction of the T1 vertebral body. Abnormal marrow intensities are seen over C3-T3, associated with an extensive collection over paravertebral and prevertebral areas of C7-T3. Subligamentous spread and epidural extension from C3 to T2 cause mass effect and spinal cord compression, leading to spinal cord oedema. There is rim enhancement of the collection with enhancement of surrounding muscle.

Further imaging with magnetic resonance imaging (MRI) revealed a loss of normal cervical lordosis with the destruction of the T1 vertebral body, abnormal marrow intensities over C3-T3, and an extensive collection over paravertebral and prevertebral areas of C7-T3 with subligamentous spread and epidural extension extending from C3 to T2. This caused mass effect and spinal cord compression with spinal cord oedema. There was rim enhancement of the collection with enhancement of surrounding muscle suggestive of infective or inflammatory changes, as depicted in Figure 3. All intervertebral discs maintained their height and signal.

Based on the clinical presentation, blood parameters, and radiological findings, a provisional diagnosis of tuberculous spondylitis of C7-T2 with an epidural abscess and upper thoracic myelopathy was made. However, a cellular, molecular, or histopathological diagnosis is needed to rule out pyogenic infection and bone metastasis pathologies before the initiation of antitubercular therapy.

Treatment

Given the diagnostic challenge surrounding the establishment of tuberculous pathology, an open biopsy of the posterior element of T2 was performed, as evident from the destruction of the spinous process of T2 on both CT scan and MRI, with infective changes surrounding the posterior paraspinal soft tissue. Intraoperative findings revealed a diseased, eroded, and softened T2 spinous process with no caseous material or pus surrounding the posterior soft tissue. Unfortunately, all cellular, molecular, and histopathological results were not suggestive of tuberculosis infection. Tests were negative for bacterial, fungal, and tuberculous cultures, negative for fast bacilli staining, and tuberculosis Gene Xpert was not detected. The histopathological findings revealed features of an inflammatory process consistent with acute-on-chronic inflammation, with no epithelioid granuloma or malignancy observed.

In pursuit of confirmatory evidence of tuberculosis infection, a discussion with the otorhinolaryngology team was initiated after a cervical lymph node examination revealed a palpable node over the submandibular and superficial anterior cervical areas. An open cervical nodes biopsy was performed, but the results were inconclusive for tuberculosis infection, showing negative cellular, molecular, and histopathological results.

Following a multidisciplinary team discussion on the patient's treatment, involving respiratory and infectious disease physicians who were reluctant to start empirical antitubercular therapy without confirmatory tests, a CT-guided lung biopsy was performed after a CT thorax revealed lung consolidation in the right upper lobe. Unfortunately, limited biopsy samples showed that tuberculosis Gene Xpert was not detected, and histopathological examination revealed acute-on-chronic inflammation with no epithelioid granuloma.

Due to the treatment delay for confirmatory tests over the past two months, a multidisciplinary team agreed to start a quadruple regimen of antitubercular therapy (isoniazid, rifampicin, ethambutol, and pyrazinamide) empirically. Close monitoring and follow-up were accomplished, and after two weeks of starting antitubercular therapy, the patient subjectively noticed a positive response to the treatment, experiencing an increase in appetite, more energy, and an improvement in general well-being. Blood parameter assessments revealed no drug toxicity and there was a downtrend in infective parameters, with a half reduction in both CRP and ESR, reading 19.5 mg/dL and 56 mm, respectively, at six weeks.

**Figure 4:**
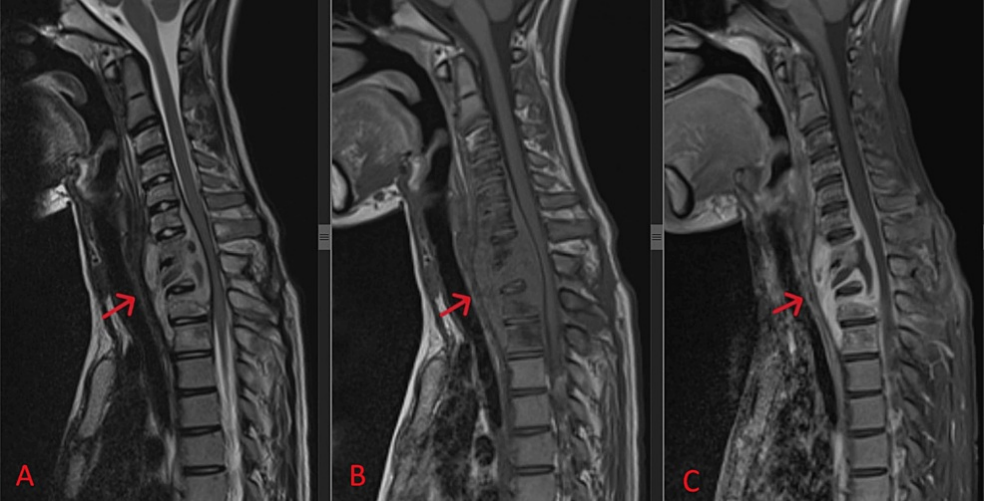
Mid-sagittal view of T2-weighted (A), T1-weighted (B), and contrast-enhanced (C) MRI of the cervicothoracic spine, with a red arrow indicating a previously seen rim-enhancing collection over paravertebral, prevertebral, and epidural spaces, now slightly smaller and exerting less mass effect on the spinal cord compared to previous imaging. There is a lesser degree of enhancement of marrow signal intensities and surrounding muscle compared to previous imaging.

An MRI reassessment at three months of antitubercular therapy (Figure 4) revealed a good response to the treatment, with evidence of a previously seen rim-enhancing collection over paravertebral, prevertebral, and epidural spaces being slightly smaller and exerting a lesser mass effect onto the spinal cord compared to previous imaging. There was a lesser degree of enhancement of marrow signal intensities and surrounding muscle compared to previous imaging.

**Figure 5:**
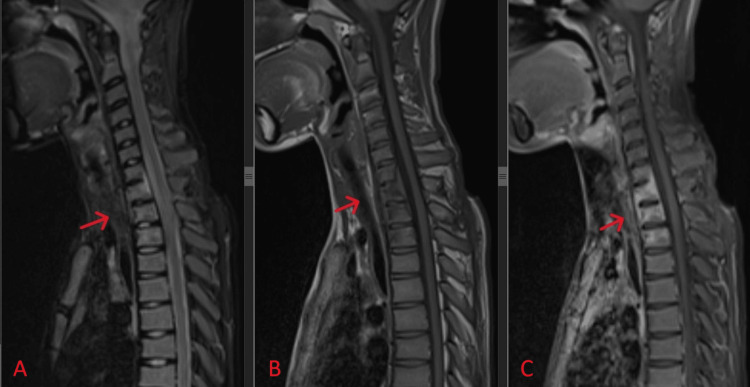
Mid-sagittal view of T2-weighted (A), T1-weighted (B), and contrast-enhanced (C) MRI of the cervicothoracic spine, with a red arrow indicating the previously seen rim-enhancement collection at the cervicothoracic junction has resolved. There is minimal residual enhancement at the body of C7 to T2 and no more mass effect on the spinal cord.

At nine months of follow-up, the patient regained normal function of the lower limbs and was able to walk independently, with resolution of the upper motor lesion deficit of bilateral lower limbs. Infective parameters normalized for both CRP and ESR. MRI reassessment showed that the previously seen rim-enhancement collection at the cervicothoracic junction had resolved, with minimal residual enhancement at the body of C7 to T2 and no more mass effect on the spinal cord. The spinal cord returned to normal signal intensity (Figure 5). The patient continued antitubercular therapy to complete the one-year course.

## Discussion

Although spinal tuberculosis is primarily a skeletal disease, secondary involvement of the nervous system may lead to diverse neurological disabilities, with the incidence of neurological complications in spinal tuberculosis ranging from 10% to 41%, as reported by Kotil et al. [3]. Considering the potentially devastating nature of the disease, a definite diagnosis is crucial for successfully treating and preventing the sequelae of tuberculosis infection while avoiding the complications of long-term antitubercular therapy.

The cornerstone of treatment for tuberculosis infection is medical treatment with antitubercular therapy. Antitubercular drugs have good penetration into vertebrae affected by tuberculosis [4]. The success of antitubercular therapy alone in the absence of surgery is high, ranging from 82% to 95%, as reported by Bakhsh [5]. Even in patients with paraplegia, recovery from pain, neurological deficit, and spinal deformity may occur in 40% of cases with medical management alone [6]. For bone and joint tuberculosis, the World Health Organization recommends extending treatment for a total of nine to 12 months due to the potentially serious nature of complications as well as difficulty in assessing response in these conditions [4].

It is pivotal for all patients to have a biopsy-proven diagnosis before starting antitubercular therapy, with multiple options for biopsies in spinal tuberculosis. The growth of *Mycobacterium* in culture specimens obtained from the infected tissue is the single most confirmatory diagnostic test for spinal tuberculosis and is considered the gold standard method. However, due to its very poor sensitivity, histopathological studies demonstrating classical granulomas and staining of smears to identify AFB are considered reference standards for all other diagnostic modalities [7]. Molecular diagnostics, such as polymerase chain reaction (PCR) and Gene Xpert MTB/RIF (*Mycobacterium tuberculosis*/rifampicin) test, are frequently used because of their rapidity and reliability with high sensitivity and specificity. In addition, they also aid in identifying resistance to rifampicin, as concluded by Maynard-Smith et al. [8].

Paraspinal abscesses and bony lesions can be sampled percutaneously by an imaging-guided approach with an imaging intensifier, ultrasound, and CT scan. Apart from that, an open biopsy can also be done for both soft tissue and bony lesions. Nevertheless, a lung biopsy can be done under the guidance of a CT scan if other biopsies do not prove tuberculosis. This case illustrates the real challenge of making a correct diagnosis of spinal tuberculosis even with appropriate clinical evaluation and investigations, including imaging, microbiological, molecular, and histological tests from multiple biopsy samples of various sites of involvement. Kumar et al. reported that this is especially true for extrapulmonary tuberculosis, which is usually paucibacillary, and manifestations are often non-specific [9].

Apart from the gold standard tissue-proven biopsy for tuberculosis infection, imaging is of immense diagnostic value for the management of spinal tuberculosis. CT scans detail skeletal involvement, and MRI is the imaging modality of choice to provide the extent of soft tissue and spinal cord involvement. Certain features of MRI findings strongly support the diagnosis of spinal tuberculosis, such as a paraspinal collection with a thin-walled abscess, a subligamentous extension of abscess beyond two vertebrae, multilevel vertebral involvement, and T2-weighted image hyperintense signal changes [10]. Despite typical and highly suggestive radiological features, none are pathognomonic, and tissue diagnosis is essential, as concluded by Garg et al. [4].

The indication for surgery in spinal tuberculosis has declined with the advent of effective antitubercular therapy. Our patient was not indicated for surgical intervention due to the absence of neurological deficits, physiological instability, spinal deformity, and a low threshold of suspicion for drug resistance, as recommended by Khanna et al. [11]. The dilemma for surgery in our case was the uncertainty of the diagnosis, and despite undergoing an open biopsy of the posterior element of T2, the diagnosis remained unproven even though the tissue sample was highly representative, as evident based on MRI findings. Based on this finding, we are inclined to repeat the biopsy with a sampling of the anterior structure of the spine and paraspinal abscess.

Since tuberculosis is a treatable condition and is associated with a poor outcome if untreated, empirical use of antitubercular therapy can be justified in cases where the index of suspicion is high, supported by typical and highly indicative radiological features [12]. However, close monitoring and follow-up are needed to confirm an appropriate clinical response, validate its further continuation, and detect drug toxicity at the earliest [7]. The empirical use of antitubercular therapy should not be equated with inappropriate and indiscriminate use. The unwarranted use of antitubercular drugs is not safe due to its propensity to cause severe hepatotoxicity and even death by causing acute liver failure, as emphasized by Kumar et al. [13].

Owing to the largely clinical and radiological nature of spinal tuberculosis diagnostics, there is an inherent risk of both overdiagnosis and underdiagnosis. In a recent study, Kumaran et al. reported that nearly 25% of patients with alternative diagnoses were radiologically reported as tuberculosis or tuberculosis formed a differential diagnosis [14]. This highlights the notion that obtaining a microbiological or pathological diagnosis may be vital, especially if radiology is not highly typical for tuberculosis, rather than empirical therapy.

This patient is an example of a good prognosis, not developing complications and experiencing relief in pain and deficits, as well as deformity, with medical therapy alone. In a study by Dunn et al., 82 patients with spinal tuberculosis were followed up. Of these, 52% were in a non-ambulatory state at presentation, and 21% had mild neurologic deficits. Among the patients with neurological deficits, 92% had significant recovery, and 74% became ambulatory from an initially non-ambulatory state with medical therapy alone [15]. Other studies in the past decade have also demonstrated good prognoses and favourable outcomes for the medical therapy of spinal tuberculosis [16,17].

## Conclusions

Spinal tuberculosis poses diagnostic as well as management challenges due to difficulties in establishing a microbiological or pathological diagnosis. The possibility of tuberculosis mimics must be carefully considered in all cases, and efforts made to rule out these possibilities. Additionally, clinical judgment and the index of suspicion for tuberculosis are critical in deciding to initiate treatment even before the tissue-proven diagnosis, with close monitoring and follow-up to ensure successful treatment. The assessment of the response to therapy is another challenge, and the diagnosis of drug-resistant spinal tuberculosis is always presumptive.
